# Woods Plastic Metallic Fillings

**Published:** 1862-11

**Authors:** 


### WOOD'S PLASTIC METALLIC FILLINGS.

We have been using this preparation for a short time, and find it very good in several particulars; it can be used any place where tin foil can, and in many places where it can not. For filling up large cavities in the molar teeth, where the use of gold is not admissable, it is the best preparation we have. The inventor does not recommend it for filling up dead teeth, yet we have in several cases cleansed out the pulp chamber and canals, forcing to the bottom of the latter pledgets of cotton moistened with creosote, filled all up nicely with this material, and all seem as yet to be doing well, and we can not see why it will not answer a good purpose, at least be far better than nothing, especially in those cases where circumstances forbid the use of gold; it can rapidly and readily be built into any required shape. We have in a number of cases used it for temporary filling, in the treatment of sensitive dentine, and particularly in the treatment of exposed nerves, when the object is to protect an exposed point till new dentine is formed, or till the conditions are so changed as to admit of a permanent filling. The cavity of decay being cleansed out, a small thin piece of spunk, saturated with creosote or spirits of camphor, or whatever may be regarded best, is placed upon the point of exposure; then pieces of metal are laid in the cavity, and with the instrument of the proper temperature it is made plastic and adapted perfectly to the walls of the cavity, the whole being filled without any disposition to move the spunk, or make pressure upon it that shall affect the pulp. This may be worn as long as desired, and then be removed as easily as it was introduced.

We have in a few cases used it in roots largely decayed, that for a time were required to retain pivot teeth. Dress out the decayed canal, forming it so that it will retain the filling, then fuse it in, dress off and drill for the pivot, and introduce the tooth to its position. We are much pleased with the cases thus far, of course we do not expect much of them in regard to duration. There is an abundant supply of it at Toland's Dental depot.

